# Regulation of TGF-β receptor activity

**DOI:** 10.1186/2045-3701-2-9

**Published:** 2012-03-15

**Authors:** Fei Huang, Ye-Guang Chen

**Affiliations:** 1 The State Key Laboratory of Biomembrane and Membrane Biotechnology, THU-PKU Joint Center for Life Sciences, School of Life Sciences, Tsinghua University, Beijing, 100084, China

**Keywords:** TGF-β receptor, phosphorylation, ubiquitination, degradation

## Abstract

TGF-β signaling regulates diverse cellular processes, including cell proliferation, differentiation, apoptosis, cell plasticity and migration. Its dysfunctions can result in various kinds of diseases, such as cancer and tissue fibrosis. TGF-β signaling is tightly regulated at different levels along the pathway, and modulation of TGF-β receptor activity is a critical step for signaling regulation. This review focuses on our recent understanding of regulation of TGF-β receptor activity.

## Introduction

Transforming growth factor-β (TGF-β) family, including TGF-β, activin, Nodal, bone morphogenetic proteins (BMPs) and others, play vital roles in development, tissue homeostasis and some diseases development [1-3]. TGF-β signaling is initiated by the binding of TGF-β to its serine and threonine kinase receptors, the type II (TβRII) and type I (TβRI) receptors on the cell membrane. Ligand binding leads to formation of the receptor heterocomplex, in which TβRII phosphorylates threonine and serine residues in the TTSGSGSG motif of TβRI and thus activates TβRI [2,4]. The activated TβRI recruits and phosphorylates the R-Smad proteins, Smad2/3 for TGF-β and activin signaling while Smad1/5/8 for BMP signaling, which then form a heterocomplex with the Co-Smad Smad4 [5,6]. The Smad complexes are then translocated into the nucleus to regulate transcription of the target genes in cooperation with other co-factors [5,7,8]. For each member of the TGF-β family, they have their own type I and type II receptors. Among the seven type I receptors, which are also called as activin receptor-like kinases (ALKs), TβRI/ALK5 can mediate TGF-β signaling with the TGF-β type II receptor TβRII to activate Smad2/3 in universal cell types, while in endothelial cells ALK1 functions with TβRII to activate Smad1/5/8 for TGF-β signaling [8-10]. In response to BMPs, ALK2/3/6 can activate Smad1/5/8 with the type II receptors BMPRII, ActRII and ActRIIB [11,12]. ALK4/7 can activate Smad2/3 with ActRII and ActRIIB to mediate activin/Nodal signaling [13]. In this review, we mainly discuss the regulation mechanisms of TGF-β signaling receptors.

In addition to activating Smad2/3, TGF-β can also activate mitogen-activating protein kinases (MAPKs) (ERK, p38 and JNK), phosphatidylinositol 3 kinase (PI3K)/Akt and small GTPases in a context-dependent manner [14-17]. Furthermore, despite the fact that TGF-β can activate Smad1/5/8 in endothelial cells which requires ALK1 [18,19], it can also activate Smad1/5/8 in other types of cells that is facilitated by the BMP type I receptors ALK2/3/6 or by other unclear mechanisms [20-22]. Those Smads are previously regarded solely as the substrates of BMP receptors to mediate BMP signaling. As modulation of the receptor activity is important for TGF-β signaling, much attention has been paid to this issue. This topic has been covered in many excellent review articles, including the one by Kang et al [23]. The current article attempts to summarize the recent development of our understanding on TGF-β receptor activity regulation.

### Phosphorylation of TGF-β receptors

**Figure 1:**
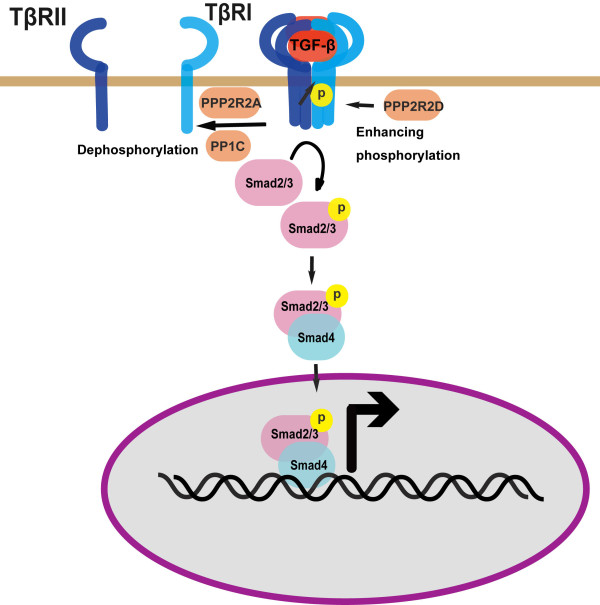
**Phosphorylation of TGF-β receptors**. Phosphorylation of TGF-β receptors regulates their activity and thus downstream Smad-dependent signaling.

Despite the fact that they are protein kinases themselves, TGF-β receptors also function as substrates for phosphorylation to regulate their activity [4] (Figure 1). TβRII are constitutively active and can undergo autophosphorylation. Ser213 and Ser409 phosphorylation are essential for TβRII's kinase activity while Ser416 phosphorylation has the inhibitory effect [24]. TβRI activation requires the phosphorylation in its GS domain (TTSGSGSG) by TβRII, and mutation of two or more residues in this motif impairs TβRI kinase activity and further disrupts expression of a Smad-dependent reporter [25]. For still unknown mechanisms, however, substitution of the non-phosphorylation residue Thr204 by aspartic acid leads to partial activation independent of ligands [25]. The residue Ser165 of TβRI can also be phosphorylated upon ligands stimulation [26]. Interestingly, substitution of Ser165 with alanine, glutamic acid or aspartic acid has no effect on TGF-β-induced reporter expression, but increases TGF-β-mediated growth inhibition and extracellular matrix formation and decreases TGF-β-induced apoptosis [26]. The same study has also identified several other phosphoserine residues, but the functional significance of these phosphorylations is unclear [26]. TGF-β receptors are thought to possess both Ser/The kinase activity and Tyr kinase activity. Indeed, TβRII has been reported to be autophosphorylated on Tyr259, Tyr336 and Tyr424, and mutation of these three residues strongly inhibits its kinase activity [27].

Phosphorylation is a reversible process. PP1c, a catalytic subunit of the protein phosphatase 1 (PP1) was reported to dephosphorylate TβRI [28] (Figure 1). TGF-β promotes the ternary complex formation of the PP1 regulatory subunit GADD34, Smad7 and TβRI, thus leading to the recruitment of PP1c via Smad7-GADD34 to the receptor complexes. PP1-mediated dephosphorylation of TβRI serves as a negative feedback mechanism to downregulate TGF-β signaling. TβRI can also be dephosphorylated by the protein phosphatase PP2A [29]. Interestingly, Bα (PPP2R2A) and Bδ (PPP2R2D), two regulatory subunits of PP2A have been shown to have opposite functions in regulation of signaling mediated by TGF-β as well as other TGF-β family members, activin and Nodal. Bα stabilizes the type I receptors of TGF-β and activin/Nodal, while Bδ inhibits receptor kinase activity for unclear mechanisms [29]. In analogy to TβRI, it is reasonable to assume that TβRII can also undergo dephosphorylation. However, the responsible phosphatases have not been reported yet.

### Regulation of TGF-β receptor ubiquitination

**Figure 2:**
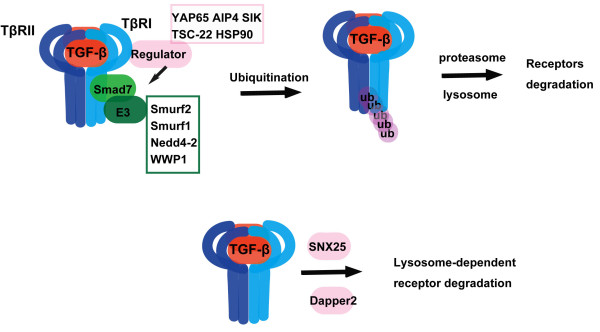
**Degradation of TGF-β receptors**. TGF-β receptors can be degraded through both ubiquitination-dependent and -independent ways. After ubiquitination, both TGF-β type I (TβRI) and type II (TβRII) receptors can be degraded via proteasome or lysosome. Although it is unclear how TβRII ubiquitination/degradation is regulated, Smad7 and Dapper2 are important adaptor proteins for ubiquitin E3 ligases (indicated in the green box)-mediated TβRI ubiquitination and degradation. Smad7-mediated ubiquitination/degradation of TβRI is finely controlled by Smad7-binding proteins indicated in the pink box.

TGF-β receptors can undergo ubiquitination-mediated degradation [30,31]. In addition to requirement of the conventional ubiquitination system containing ubiquitin E1, E2 and E3 ligases, ubiquitination of TβRI appears to need an adaptor protein, Smad7 [32]. Smad7, a member of the I-Smads, can interact with the activated TβRI and recruit the HECT domain-containing E3 ligases Smurf1, Smurf2, NEDD4-2, or WWP1 to the receptor, leading to ubiquitination and degradation of the receptor [33-37] (Figure 2).

Ubiquitination of TβRI is finely controlled by multiple proteins and mechanisms. The Salt-inducible kinase (SIK) has been reported to promote Smad7-TβRI complex formation and enhance the ubiquitination-dependent degradation of TβRI [38]. In addition, SIK is a direct transcriptional target of TGF-β signaling, and therefore it functions as a negative regulating feedback mechanism to limit TGF-β signaling [38]. Atrophin1-interacting protein 4 (AIP4) and Yes-associated protein 65 (YAP65) have been shown to enhance recruitment of Smad7 to TβRI and thus inhibit TGF-β signaling [39,40]. In contrast, several other proteins have been demonstrated to inhibit the Smad7-dependent ubiquitination of TβRI. The 90-kDa heat-shock protein (HSP90) interacts with both TβRI and TβRII, and inhibition of HSP90 activity increases Smad7/Smurf2-dependent ubiquitination of TβRI and decreases TGF-β-induced signaling [41]. TGF-β-stimulated clone 22 (TSC-22) can disrupt the binding of Smad7/Smurfs to TβRI and therefore decrease the ubiquitination and degradation of the receptor, resulting in enhanced TGF-β signaling [42]. Regulation of ubiquitination-dependent degradation of the receptors is an important aspect in termination of TGF-β signal transduction.

It seems that TGF-β receptors can be degraded in both the proteasome and lysosome pathways, and the lysosomal degradation may not always require ubiquitinaiton. For instance, Dapper2 can interact with TβRI in the Rab7-positive late endosomes and facilitate its transport to lysosomes for degradation [43,44]. It is unclear whether Smad7 and ubiquitination play any roles in this process (Figure 2). Sorting nexin 25 (SNX25) has been reported to enhance TβRI degradation in lysosomes independent of ubiquitination [45]. Although the regulation of TβRI degradation has caught reasonable attention, how TβRII degradation is regulated is less studied.

### Regulation of the heterocomplex formation of TGF-β receptors and Smad recruitment

**Figure 3:**
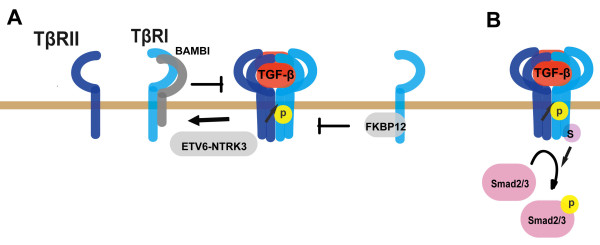
**Various mechanisms regulate TGF-β receptor activity**. (A) Regulation of TGF-β receptor activity by multiple receptor-binding proteins. BAMBI and ETV6-NTRK3 attenuates TGF-β receptor activity by interfering receptor heterocomplex formation, while FKBP12 blocks GS domain phosphorylation of TβRI by TβRII at the basal state. (B) Sumoylation promotes TβRI activity.

The tetrameric complex formation between TβRI and TβRII is essential for TGF-β signal transduction [46]. It has long been regarded that both TβRI and TβRII exist as a pre-formed dimer on the plasma membrane and ligands binding promotes the homo-dimer to form a hetero-tetramer [47-49]. However, using single-molecule imaging combined with total internal reflection fluorescence microscopy technology, TGF-β receptors were found to exist as monomers on the membrane in resting cells and undergo dimerization upon TGF-β stimulation [50,51]. Therefore, regulation of receptor complex formation is an important mechanism to control TGF-β signaling. The TGF-β coreceptor betaglycan facilitates TGF-β signaling by helping presentation of the ligands to TβRII [52-55]. However, in some cell types such as pig kidney LLC-PK1 cells, betaglycan can inhibit TGF-β heteromeric receptor complex formation to negatively regulate the signaling, indicating that betaglycan regulates TGF-β signaling at receptor level in a cell type dependent manner [53]. BMP and activin membrane-bound inhibitor (BAMBI) and the ETV6-NTRK3 chimeric tyrosine kinase have been demonstrated to attenuate TGF-β signaling by interfering with the heterocomplex formation of TGF-β receptors [56-58]. In contrast, the immunophilin FKBP12, which physically binds to the GS domain of TβRI, does not interrupt receptor complex formation, but blocks TβRI activation by TβRII [59-61](Figure 3A).

After phosphorylated by TβRII at the GS domain, TβRI is activated to interact with and phosphorylate Smad2/3. Various proteins associated with receptors complex have been reported to regulate Smad recruitment, which was already summarized in the review of Kang et al, such as SARA, STRAP and Axin [23]. Here we take SARA as an example. Smad archor for receptor activation protein (SARA), a FYVE domain protein which associates with membrane via binding to phosphatidylinosital-3 phosphate, helps recruitment of Smad2/3 to the activated TβRI to facilitate Smad activation [62]. In addition to affecting receptor complex formation, BAMBI can form a ternary complex with TβRI and Smad7 to disrupt the interactions between TβRI and Smad3 [58]. Post-translational modification of the receptors can also influence Smad recruitment. Sumoylation is a ubiquitin-like modification and regulates protein localization and activity [63]. The phosphorylated TβRI can be sumoylated at Lys389 [64]. Sumoylation of TβRI can enhance TGF-β signaling by promoting recruitment and phosphorylation of Smad3 (Figure 3B).

### Activation of MAPKs and Smad1/5/8

**Figure 4:**
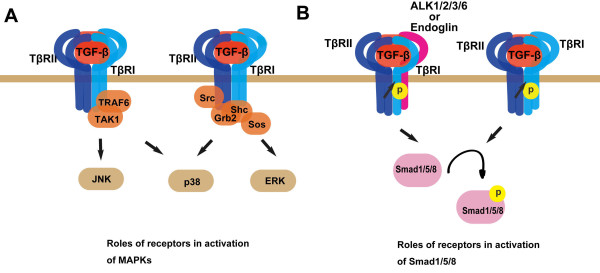
**Roles of TGF-β receptors in activation of MAPKs and Smad1/5/8**. In addition to activating Smad2/3, TGF-β can also turn on MAPKs and Smad1/5/8. (A) Activation of MAPKs can be achieved via the TRAF6-TAK1 axis or the Grb2/Shc-Ras axis. (B) In addition to TGF-β receptors, activation of Smad1/5/8 has been shown to be dependent on ALK1 and endoglin in endothelial cells or the BMP type I receptors ALK2/3/6 in other cell types.

TGF-β not only transduces its signal via Smad proteins, but can also activate other signaling molecules such as MAPKs in a cell type-specific manner (Figure 4A). Receptor activity is also required for the later event as inhibition of TβRI activity blocks TGF-β-induced MAPK activation [65]. Several studies suggested that TGF-β-mediated MAPK activation is associated with tyrosine phosphorylation of TGF-β receptors. Src was reported to phosphorylate TβRII on Tyr284 and recruit the SH2-containing adaptors Grb2 and Shc to the receptor [66]. This event may play an important role in TGF-β-mediated p38 activation although it has no effect on the canonical Smad2/3 signaling. Like TβRII, TβRI is also a dual-specificity kinase. TGF-β can induce tyrosine phosphorylation of TβRI and then phosphorylation on both tyrosine and serine residues of Shc, leading to recruitment of Grb2 and Sos, a guanine nucleotide exchange factor for Ras, and thus MAPK activation [67]. TβRI was also reported to interact with an E3 ubiquitin ligase TRAF6, which functions to mediate the activation of p38 and JNK by TGF-β [65,68]. TβRI enhances the K63-linked ubiquitination of TRAF6, leading to the activation of TAK1 and stimulation of p38 and JNK signaling.

Smad1/5/8 is usually activated by BMP, but can also be activated by TGF-β [10,18,20-22,69] (Figure 4B). It has been known that TGF-β can activate Smad1/5/8 via its endothelial-specific type I receptor ALK1 in endothelial cells [10,18]. A recent study reported that TβRI-mediated phosphorylation of endoglin, an endothelial-specific TGF-β coreceptor, is essential for TGF-β activation of Smad1/5/8 in endothelial cells [70]. In other cell types, TGF-β-mediated activation of Smad1/5/8 can be achieved via the interaction of TβRI with BMP receptors ALK2/3/6 [20], or in BMP receptor-independent mechanisms [22]. Other proteins may be involved in this process. For example, ERBB2, an EGFR family member, has been indicated in Smad1/5/8 activation induced by TGF-β [21], but the detailed mechanism still need to be defined.

### Other non-canonical TGF-β receptor functions

**Figure 5:**
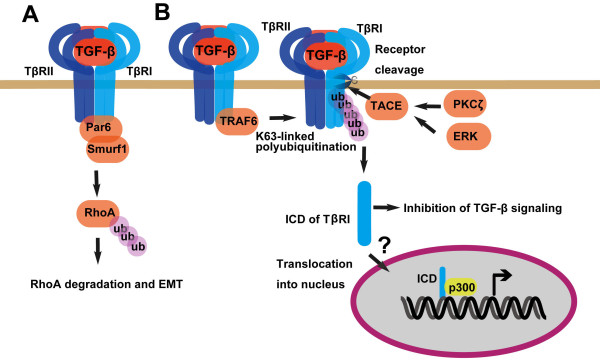
**TGF-β receptors can function independent of downstream signaling molecules Smads, MAPKs or Akt**. (A) TβRII can interact with Par6 to induce degradation of RhoA, leading to epithelia-mesenchymal transition. (B) The intracellular domain (ICD) of TβRI can be cleaved by TACE and translocate into the nucleus to regulate transcription associated with p300.

As many other cell surface receptors, TGF-β receptors mainly function through activating downstream signaling molecules, such as Smads, MAPKs and Akt in the case of TGF-β. However, it has been found that TGF-β receptors can also transduce signals via atypical manners. For instance, TβRII can interact with and phosphorylate Par6, which recruits the ubiquitin E3 ligase Smurf1 to degrade RhoA, leading to loss of tight junctions and epithelial-mesenchymal transition [71] (Figure 5A).

A recent report revealed a nuclear function of TβRI [72]. K63-linked polyubiquitination of TβRI by TRAF6 promotes its cleavage at the residue G120 by TNF-α converting enzyme TACE (Figure 5B). The released intracellular domain (TβRI-ICD) enters the nucleus and associates with p300 to regulate the expression of target genes such as Snail and MMP2. TGF-β can activate PKCζ in a TRAF6-dependent manner, and PKCζ in turn facilitates the TACE-mediated cleavage of TβRI. Blockage of the TβRI-ICD releasing attenuates TGF-β-induced invasiveness of breast MDA-MD-231 and lung A549 carcinoma cells. Interestingly, TACE, activated by ERK signaling, induced cleavage of TβRI was also shown to reduce the cell surface receptor amount and negatively regulate TGF-β signaling on anti-proliferation and epithelial-mesenchymal transition [73]. Further investigation is needed to solve these contradictory issues.

### Membrane trafficking regulates TGF-β receptor activity

**Figure 6:**
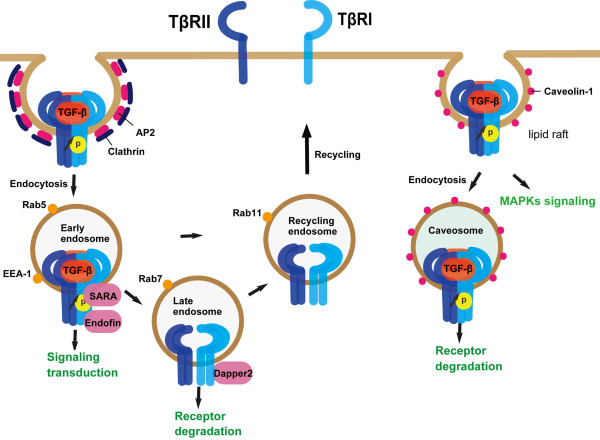
**Membrane trafficking regulates TGF-β receptor activity and degradation**. Receptors can be directed to transduce signals, be degraded or recycled by membrane trafficking.

TGF-β receptors are constitutively internalized via clathrin-dependent or lipid-raft-dependent endocytic pathways [74-76] (Figure 6). Clathrin-dependent endocytosis of the receptors has been regarded to positively facilitate TGF-β signaling while lipid raft/caveolae-mediated internalization has an inhibitory effect [77-81]. Internalization of TGF-β receptors through clathrin-dependent endocytosis to EEA1-positive endosomes is more likely to promote signaling as SARA and endofin are enriched in EEA1-positive endosomes and can facilitate R-Smads activation and Smad complex formation [78,82,83] (Figure 6). The internalized receptors are targeted to distinct destinations, and these processes are regulated by different Rab GTPases. The internalized receptors can be recycled and return to the membrane via Rab11-dependent manner [84]. The clathrin adaptor protein Dab2 was reported to target TβRII to the recycling pathway in Rab11-positive endosomes [85]. Once the receptors are transported to Rab7-positive later endosomes, Dapper2 can associate with activated TβRI and direct it to lysosome for degradation [43].

TGF-β receptors are partitioned between the lipid raft microdomains and non-raft parts on the plasma membrane [86-91], and the partitioning has been shown to be regulated [74,87]. Caveolin-1, a protein enriched in caveolae, inhibits TGF-β signaling by interacting with TβRI [92] and promotes TβRI degradation in a Smad7/Smurf2-dependent manner [78]. Caveolin-1-mediated TGF-β receptor degradation is enhanced by CD109, a GPI-anchored protein that can function as a TGF-β co-receptor [93,94]. Distribution of TGF-β receptors in lipid rafts does not simply promote receptor degradation, it is also required for TGF-β-mediated MAPK activation [95] (Figure 6). Disturbance of distribution of TGF-β receptors in lipid rafts by cholesterol depletion blocks TGF-β-induced MAPK activation and epithelial-mesenchymal transition.

## Conclusions and Perspectives

Modulation of receptor activity is a critical step for TGF-β signaling regulation. Although much effort has been made to understand the regulatory mechanisms of TGF-β receptor activity and stability, many questions still await to be addressed. Ubiquitination is known to promote TGF-β receptor degradation. However, its role in mediating TGF-β receptor endocytosis is unclear. Although membrane trafficking of the receptors has been quite extensively investigated, it is still far from establishment of the complete picture. Furthermore, how ubiquitination regulates TβRII is less understood, and the ubiquitin E3 ligases for TβRII are still missing. Although TGF-β receptors are found to be modified by phosphorylation, ubiqitination and sumoylation, whether the receptors also undergo other post-translational modifications, such as acetylation, neddylation, PARylation and others is still an open question. In addition to the canonical activity as a kinase, TGF-β receptors have also been suggested to have other functions. For instance, the cleaved intracellular domain of TβRI has transcriptional activation activity in the nucleus. It remains to test whether TβRII has a similar function. Moreover, it has been reported that TGF-β-mediated activation of ERK in human skins is dependent on TβRII, but not TβRI [96], re-raising the question whether the two types of TGF-β receptors can activate noncanonical signaling pathways independently of each other through new mechanisms. Histone acetylation has been indicated to regulate TGF-β receptor expression [97-100]. Other mechanisms may be also employed to control their transcription. For instance, microRNA mir-106b has been reported to repress TβRII expressions [101], and the activin type I receptor ALK4 is a target of mir-24 [102]. Therefore, exploring the molecular mechanisms of how the TGF-β receptor activity is modulated will still be an exciting field.

## List of abbreviations

AIP4: Atrophin1-interacting protein 4; ALK1/2/3/4/6: activin A receptor type II-like 1/2/3/5/6; BAMBI: activin membrane-bound inhibitor; BMP: bone morphogenetic protein; EN: ETV6-NTRK3 chimeric tyrosine kinase; HSP90: 90-kDa heat-shock protein; MAPK: mitogen-activating protein kinase; PI3K: phosphatidylinositol 3 kinase; SARA: Smad archor for receptor activation; SIK: Salt-inducible kinase; SNX25: sorting nexin 25; STRAP: serine/threonine kinase receptor associated protein; TβRI: transforming growth factor-β receptor type I; TβRII: transforming growth factor-β receptor type II; TGF-β: transforming growth factor-β; TRAF6: TNF receptor-associated factor 6; TACE: TNF-α converting enzyme; TSC-22 (TGF-β-stimulated clone 22); YAP65: Yes-associated protein 65.

## Competing interests

The authors declare that they have no competing interests.

## Authors' contributions

FH and YGC wrote the manuscript. All authors read and approved the final manuscript.

## References

[B1] Massague J Blain SW Lo RS TGFbeta signaling in growth control, cancer, and heritable disorders Cell 2000 103 295 309 10.1016/S0092-8674(00)00121-5 11057902

[B2] Massague J Chen YG Controlling TGF-beta signaling Genes Dev 2000 14 627 644 10733523

[B3] Schmierer B Hill CS TGFbeta-SMAD signal transduction: molecular specificity and functional flexibility Nat Rev Mol Cell Biol 2007 8 970 982 10.1038/nrm2297 18000526

[B4] Wrighton KH Lin X Feng XH Phospho-control of TGF-beta superfamily signaling Cell Res 2009 19 8 20 10.1038/cr.2008.327 19114991 PMC2929013

[B5] Chen X Xu L Mechanism and Regulation of Nucleocytoplasmic Trafficking of Smad Cell Biosci 2011 1 40 10.1186/2045-3701-1-40 22204445 PMC3292837

[B6] Tang LY Zhang YE Non-degradative ubiquitination in Smad-dependent TGF-beta signaling Cell Biosci 2011 1 43 10.1186/2045-3701-1-43 22204598 PMC3293007

[B7] Massague J Seoane J Wotton D Smad transcription factors Genes Dev 2005 19 2783 2810 10.1101/gad.1350705 16322555

[B8] Feng XH Derynck R Specificity and versatility in tgf-beta signaling through Smads Annu Rev Cell Dev Biol 2005 21 659 693 10.1146/annurev.cellbio.21.022404.142018 16212511

[B9] Lebrin F Goumans MJ Jonker L Carvalho RL Valdimarsdottir G Thorikay M Mummery C Arthur HM ten Dijke P Endoglin promotes endothelial cell proliferation and TGF-beta/ALK1 signal transduction EMBO J 2004 23 4018 4028 10.1038/sj.emboj.7600386 15385967 PMC524335

[B10] Goumans MJ Valdimarsdottir G Itoh S Lebrin F Larsson J Mummery C Karlsson S ten Dijke P Activin receptor-like kinase (ALK)1 is an antagonistic mediator of lateral TGFbeta/ALK5 signaling Mol Cell 2003 12 817 828 10.1016/S1097-2765(03)00386-1 14580334

[B11] Sieber C Kopf J Hiepen C Knaus P Recent advances in BMP receptor signaling Cytokine Growth Factor Rev 2009 20 343 355 10.1016/j.cytogfr.2009.10.007 19897402

[B12] Miyazono K Maeda S Imamura T BMP receptor signaling: transcriptional targets, regulation of signals, and signaling cross-talk Cytokine Growth Factor Rev 2005 16 251 263 10.1016/j.cytogfr.2005.01.009 15871923

[B13] Walton KL Makanji Y Harrison CA New insights into the mechanisms of activin action and inhibition Mol Cell Endocrinol 2011 10.1016/j.mce.2011.06.030 21763751

[B14] Moustakas A Heldin CH Non-Smad TGF-beta signals J Cell Sci 2005 118 3573 3584 10.1242/jcs.02554 16105881

[B15] Derynck R Zhang YE Smad-dependent and Smad-independent pathways in TGF-beta family signalling Nature 2003 425 577 584 10.1038/nature02006 14534577

[B16] Zhang YE Non-Smad pathways in TGF-beta signaling Cell Res 2009 19 128 139 10.1038/cr.2008.328 19114990 PMC2635127

[B17] Chapnick DA Warner L Bernet J Rao T Liu X Partners in Crime: TGF-beta and MAPK pathways in cancer progression Cell Biosci 2011 1 42 10.1186/2045-3701-1-42 22204556 PMC3275500

[B18] Pardali E Goumans MJ ten Dijke P Signaling by members of the TGF-beta family in vascular morphogenesis and disease Trends Cell Biol 2010 20 556 567 10.1016/j.tcb.2010.06.006 20656490

[B19] Lebrin F Deckers M Bertolino P Ten Dijke P TGF-beta receptor function in the endothelium Cardiovasc Res 2005 65 599 608 10.1016/j.cardiores.2004.10.036 15664386

[B20] Daly AC Randall RA Hill CS Transforming growth factor beta-induced Smad1/5 phosphorylation in epithelial cells is mediated by novel receptor complexes and is essential for anchorage-independent growth Mol Cell Biol 2008 28 6889 6902 10.1128/MCB.01192-08 18794361 PMC2573298

[B21] Liu IM Schilling SH Knouse KA Choy L Derynck R Wang XF TGFbeta-stimulated Smad1/5 phosphorylation requires the ALK5 L45 loop and mediates the pro-migratory TGFbeta switch EMBO J 2009 28 88 98 10.1038/emboj.2008.266 19096363 PMC2634733

[B22] Wrighton KH Lin X Yu PB Feng XH Transforming Growth Factor {beta} Can Stimulate Smad1 Phosphorylation Independently of Bone Morphogenic Protein Receptors J Biol Chem 2009 284 9755 9763 10.1074/jbc.M809223200 19224917 PMC2665096

[B23] Kang JS Liu C Derynck R New regulatory mechanisms of TGF-beta receptor function Trends Cell Biol 2009 19 385 394 10.1016/j.tcb.2009.05.008 19648010

[B24] Luo K Lodish HF Positive and negative regulation of type II TGF-beta receptor signal transduction by autophosphorylation on multiple serine residues EMBO J 1997 16 1970 1981 10.1093/emboj/16.8.1970 9155023 PMC1169800

[B25] Wieser R Wrana JL Massague J GS domain mutations that constitutively activate T beta R-I, the downstream signaling component in the TGF-beta receptor complex EMBO J 1995 14 2199 2208 7774578 10.1002/j.1460-2075.1995.tb07214.x PMC398326

[B26] Souchelnytskyi S ten Dijke P Miyazono K Heldin CH Phosphorylation of Ser165 in TGF-beta type I receptor modulates TGF-beta1-induced cellular responses EMBO J 1996 15 6231 6240 8947046 PMC452446

[B27] Lawler S Feng XH Chen RH Maruoka EM Turck CW Griswold-Prenner I Derynck R The type II transforming growth factor-beta receptor autophosphorylates not only on serine and threonine but also on tyrosine residues J Biol Chem 1997 272 14850 14859 10.1074/jbc.272.23.14850 9169454

[B28] Shi W Sun C He B Xiong W Shi X Yao D Cao X GADD34-PP1c recruited by Smad7 dephosphorylates TGFbeta type I receptor J Cell Biol 2004 164 291 300 10.1083/jcb.200307151 14718519 PMC2172339

[B29] Batut J Schmierer B Cao J Raftery LA Hill CS Howell M Two highly related regulatory subunits of PP2A exert opposite effects on TGF-beta/Activin/Nodal signalling Development 2008 135 2927 2937 10.1242/dev.020842 18697906 PMC4940033

[B30] Itoh S ten Dijke P Negative regulation of TGF-beta receptor/Smad signal transduction Curr Opin Cell Biol 2007 19 176 184 10.1016/j.ceb.2007.02.015 17317136

[B31] Lonn P Moren A Raja E Dahl M Moustakas A Regulating the stability of TGFbeta receptors and Smads Cell Res 2009 19 21 35 10.1038/cr.2008.308 19030025

[B32] Yan X Chen YG Smad7: not only a regulator, but also a cross-talk mediator of TGF-beta signalling Biochem J 2011 434 1 10 10.1042/BJ20101827 21269274

[B33] Ebisawa T Fukuchi M Murakami G Chiba T Tanaka K Imamura T Miyazono K Smurf1 interacts with transforming growth factor-beta type I receptor through Smad7 and induces receptor degradation J Biol Chem 2001 276 12477 12480 10.1074/jbc.C100008200 11278251

[B34] Hayashi H Abdollah S Qiu Y Cai J Xu YY Grinnell BW Richardson MA Topper JN Gimbrone MA Jr Wrana JL Falb D The MAD-related protein Smad7 associates with the TGFbeta receptor and functions as an antagonist of TGFbeta signaling Cell 1997 89 1165 1173 10.1016/S0092-8674(00)80303-7 9215638

[B35] Kavsak P Rasmussen RK Causing CG Bonni S Zhu H Thomsen GH Wrana JL Smad7 binds to Smurf2 to form an E3 ubiquitin ligase that targets the TGF beta receptor for degradation Mol Cell 2000 6 1365 1375 10.1016/S1097-2765(00)00134-9 11163210

[B36] Kuratomi G Komuro A Goto K Shinozaki M Miyazawa K Miyazono K Imamura T NEDD4-2 (neural precursor cell expressed, developmentally down-regulated 4-2) negatively regulates TGF-beta (transforming growth factor-beta) signalling by inducing ubiquitin-mediated degradation of Smad2 and TGF-beta type I receptor Biochem J 2005 386 461 470 10.1042/BJ20040738 15496141 PMC1134864

[B37] Komuro A Imamura T Saitoh M Yoshida Y Yamori T Miyazono K Miyazawa K Negative regulation of transforming growth factor-beta (TGF-beta) signaling by WW domain-containing protein 1 (WWP1) Oncogene 2004 23 6914 6923 10.1038/sj.onc.1207885 15221015

[B38] Kowanetz M Lonn P Vanlandewijck M Kowanetz K Heldin CH Moustakas A TGFbeta induces SIK to negatively regulate type I receptor kinase signaling J Cell Biol 2008 182 655 662 10.1083/jcb.200804107 18725536 PMC2518705

[B39] Lallemand F Seo SR Ferrand N Pessah M L'Hoste S Rawadi G Roman-Roman S Camonis J Atfi A AIP4 restricts transforming growth factor-beta signaling through a ubiquitination-independent mechanism J Biol Chem 2005 280 27645 27653 10.1074/jbc.M500188200 15946939

[B40] Ferrigno O Lallemand F Verrecchia F L'Hoste S Camonis J Atfi A Mauviel A Yes-associated protein (YAP65) interacts with Smad7 and potentiates its inhibitory activity against TGF-beta/Smad signaling Oncogene 2002 21 4879 4884 10.1038/sj.onc.1205623 12118366

[B41] Wrighton KH Lin X Feng XH Critical regulation of TGFbeta signaling by Hsp90 Proc Natl Acad Sci USA 2008 105 9244 9249 10.1073/pnas.0800163105 18591668 PMC2453700

[B42] Yan X Zhang J Pan L Wang P Xue H Zhang L Gao X Zhao X Ning Y Chen YG TSC-22 promotes transforming growth factor beta-mediated cardiac myofibroblast differentiation by antagonizing Smad7 activity Mol Cell Biol 2011 31 3700 3709 10.1128/MCB.05448-11 21791611 PMC3165719

[B43] Zhang L Zhou H Su Y Sun Z Zhang H Zhang L Zhang Y Ning Y Chen YG Meng A Zebrafish Dpr2 inhibits mesoderm induction by promoting degradation of nodal receptors Science 2004 306 114 117 10.1126/science.1100569 15459392

[B44] Su Y Zhang L Gao X Meng F Wen J Zhou H Meng A Chen YG The evolutionally conserved activity of Dapper2 in antagonizing TGF-beta signaling FASEB J 2007 21 682 690 10.1096/fj.06-6246com 17197390

[B45] Hao X Wang Y Ren F Zhu S Ren Y Jia B Li YP Shi Y Chang Z SNX25 regulates TGF-beta signaling by enhancing the receptor degradation Cell Signal 2011 23 935 946 10.1016/j.cellsig.2011.01.022 21266196

[B46] Wrana JL Attisano L Wieser R Ventura F Massague J Mechanism of activation of the TGF-beta receptor Nature 1994 370 341 347 10.1038/370341a0 8047140

[B47] Chen RH Derynck R Homomeric interactions between type II transforming growth factor-beta receptors J Biol Chem 1994 269 22868 22874 7521335

[B48] Gilboa L Wells RG Lodish HF Henis YI Oligomeric structure of type I and type II transforming growth factor beta receptors: homodimers form in the ER and persist at the plasma membrane J Cell Biol 1998 140 767 777 10.1083/jcb.140.4.767 9472030 PMC2141740

[B49] Henis YI Moustakas A Lin HY Lodish HF The types II and III transforming growth factor-beta receptors form homo-oligomers J Cell Biol 1994 126 139 154 10.1083/jcb.126.1.139 8027173 PMC2120107

[B50] Zhang W Jiang Y Wang Q Ma X Xiao Z Zuo W Fang X Chen YG Single-molecule imaging reveals transforming growth factor-beta-induced type II receptor dimerization Proc Natl Acad Sci USA 2009 106 15679 15683 10.1073/pnas.0908279106 19720988 PMC2747179

[B51] Zhang W Yuan J Yang Y Xu L Wang Q Zuo W Fang X Chen YG Monomeric type I and type III transforming growth factor-beta receptors and their dimerization revealed by single-molecule imaging Cell Res 2010 20 1216 1223 10.1038/cr.2010.105 20625381

[B52] Lopez-Casillas F Wrana JL Massague J Betaglycan presents ligand to the TGF beta signaling receptor Cell 1993 73 1435 1444 10.1016/0092-8674(93)90368-Z 8391934

[B53] Eickelberg O Centrella M Reiss M Kashgarian M Wells RG Betaglycan inhibits TGF-beta signaling by preventing type I-type II receptor complex formation. Glycosaminoglycan modifications alter betaglycan function J Biol Chem 2002 277 823 829 11668175 10.1074/jbc.M105110200

[B54] Esparza-Lopez J Montiel JL Vilchis-Landeros MM Okadome T Miyazono K Lopez-Casillas F Ligand binding and functional properties of betaglycan, a co-receptor of the transforming growth factor-beta superfamily. Specialized binding regions for transforming growth factor-beta and inhibin A J Biol Chem 2001 276 14588 14596 10.1074/jbc.M008866200 11278442

[B55] Bilandzic M Stenvers KL Betaglycan: a multifunctional accessory Mol Cell Endocrinol 2011 339 180 189 10.1016/j.mce.2011.04.014 21550381

[B56] Onichtchouk D Chen YG Dosch R Gawantka V Delius H Massague J Niehrs C Silencing of TGF-beta signalling by the pseudoreceptor BAMBI Nature 1999 401 480 485 10.1038/46794 10519551

[B57] Jin W Kim BC Tognon C Lee HJ Patel S Lannon CL Maris JM Triche TJ Sorensen PH Kim SJ The ETV6-NTRK3 chimeric tyrosine kinase suppresses TGF-beta signaling by inactivating the TGF-beta type II receptor Proc Natl Acad Sci USA 2005 102 16239 16244 10.1073/pnas.0503137102 16258068 PMC1283417

[B58] Yan X Lin Z Chen F Zhao X Chen H Ning Y Chen YG Human BAMBI cooperates with Smad7 to inhibit transforming growth factor-beta signaling J Biol Chem 2009 284 30097 30104 10.1074/jbc.M109.049304 19758997 PMC2781564

[B59] Chen YG Liu F Massague J Mechanism of TGFbeta receptor inhibition by FKBP12 EMBO J 1997 16 3866 3876 10.1093/emboj/16.13.3866 9233797 PMC1170011

[B60] Huse M Muir TW Xu L Chen YG Kuriyan J Massague J The TGF beta receptor activation process: an inhibitor- to substrate-binding switch Mol Cell 2001 8 671 682 10.1016/S1097-2765(01)00332-X 11583628

[B61] Huse M Chen YG Massague J Kuriyan J Crystal structure of the cytoplasmic domain of the type I TGF beta receptor in complex with FKBP12 Cell 1999 96 425 436 10.1016/S0092-8674(00)80555-3 10025408

[B62] Tsukazaki T Chiang TA Davison AF Attisano L Wrana JL SARA, a FYVE domain protein that recruits Smad2 to the TGFbeta receptor Cell 1998 95 779 791 10.1016/S0092-8674(00)81701-8 9865696

[B63] Geiss-Friedlander R Melchior F Concepts in sumoylation: a decade on Nat Rev Mol Cell Biol 2007 8 947 956 10.1038/nrm2293 18000527

[B64] Kang JS Saunier EF Akhurst RJ Derynck R The type I TGF-beta receptor is covalently modified and regulated by sumoylation Nat Cell Biol 2008 10 654 664 10.1038/ncb1728 18469808 PMC2649123

[B65] Sorrentino A Thakur N Grimsby S Marcusson A von Bulow V Schuster N Zhang S Heldin CH Landstrom M The type I TGF-beta receptor engages TRAF6 to activate TAK1 in a receptor kinase-independent manner Nat Cell Biol 2008 10 1199 1207 10.1038/ncb1780 18758450

[B66] Galliher AJ Schiemann WP Src phosphorylates Tyr284 in TGF-beta type II receptor and regulates TGF-beta stimulation of p38 MAPK during breast cancer cell proliferation and invasion Cancer Res 2007 67 3752 3758 10.1158/0008-5472.CAN-06-3851 17440088

[B67] Lee MK Pardoux C Hall MC Lee PS Warburton D Qing J Smith SM Derynck R TGF-beta activates Erk MAP kinase signalling through direct phosphorylation of ShcA EMBO J 2007 26 3957 3967 10.1038/sj.emboj.7601818 17673906 PMC1994119

[B68] Yamashita M Fatyol K Jin C Wang X Liu Z Zhang YE TRAF6 mediates Smad-independent activation of JNK and p38 by TGF-beta Mol Cell 2008 31 918 924 10.1016/j.molcel.2008.09.002 18922473 PMC2621323

[B69] Sapkota G Alarcon C Spagnoli FM Brivanlou AH Massague J Balancing BMP signaling through integrated inputs into the Smad1 linker Mol Cell 2007 25 441 454 10.1016/j.molcel.2007.01.006 17289590

[B70] Ray BN Lee NY How T Blobe GC ALK5 phosphorylation of the endoglin cytoplasmic domain regulates Smad1/5/8 signaling and endothelial cell migration Carcinogenesis 2010 31 435 441 10.1093/carcin/bgp327 20042635 PMC2832549

[B71] Ozdamar B Bose R Barrios-Rodiles M Wang HR Zhang Y Wrana JL Regulation of the polarity protein Par6 by TGFbeta receptors controls epithelial cell plasticity Science 2005 307 1603 1609 10.1126/science.1105718 15761148

[B72] Mu Y Sundar R Thakur N Ekman M Gudey SK Yakymovych M Hermansson A Dimitriou H Bengoechea-Alonso MT Ericsson J TRAF6 ubiquitinates TGFbeta type I receptor to promote its cleavage and nuclear translocation in cancer Nat Commun 2011 2 330 21629263 10.1038/ncomms1332 PMC3113296

[B73] Liu C Xu P Lamouille S Xu J Derynck R TACE-mediated ectodomain shedding of the type I TGF-beta receptor downregulates TGF-beta signaling Mol Cell 2009 35 26 36 10.1016/j.molcel.2009.06.018 19595713 PMC2740991

[B74] Chen YG Endocytic regulation of TGF-beta signaling Cell Res 2009 19 58 70 10.1038/cr.2008.315 19050695

[B75] Le Roy C Wrana JL Clathrin- and non-clathrin-mediated endocytic regulation of cell signalling Nat Rev Mol Cell Biol 2005 6 112 126 10.1038/nrm1571 15687999

[B76] Kardassis D Murphy C Fotsis T Moustakas A Stournaras C Control of transforming growth factor beta signal transduction by small GTPases Febs J 2009 276 2947 2965 10.1111/j.1742-4658.2009.07031.x 19490100

[B77] Anders RA Dore JJ Jr Arline SL Garamszegi N Leof EB Differential requirement for type I and type II transforming growth factor beta receptor kinase activity in ligand-mediated receptor endocytosis J Biol Chem 1998 273 23118 23125 10.1074/jbc.273.36.23118 9722540

[B78] Di Guglielmo GM Le Roy C Goodfellow AF Wrana JL Distinct endocytic pathways regulate TGF-beta receptor signalling and turnover Nat Cell Biol 2003 5 410 421 10.1038/ncb975 12717440

[B79] Yao D Ehrlich M Henis YI Leof EB Transforming growth factor-beta receptors interact with AP2 by direct binding to beta2 subunit Mol Biol Cell 2002 13 4001 4012 10.1091/mbc.02-07-0104 12429842 PMC133610

[B80] Lu Z Murray JT Luo W Li H Wu X Xu H Backer JM Chen YG Transforming growth factor beta activates Smad2 in the absence of receptor endocytosis J Biol Chem 2002 277 29363 29368 10.1074/jbc.M203495200 12034739

[B81] Hayes S Chawla A Corvera S TGF beta receptor internalization into EEA1-enriched early endosomes: role in signaling to Smad2 J Cell Biol 2002 158 1239 1249 10.1083/jcb.200204088 12356868 PMC2173232

[B82] Hu Y Chuang JZ Xu K McGraw TG Sung CH SARA, a FYVE domain protein, affects Rab5-mediated endocytosis J Cell Sci 2002 115 4755 4763 10.1242/jcs.00177 12432064 PMC3899687

[B83] Chen YG Wang Z Ma J Zhang L Lu Z Endofin, a FYVE domain protein, interacts with Smad4 and facilitates transforming growth factor-beta signaling J Biol Chem 2007 282 9688 9695 10.1074/jbc.M611704200 17272273

[B84] Mitchell H Choudhury A Pagano RE Leof EB Ligand-dependent and -independent transforming growth factor-beta receptor recycling regulated by clathrin-mediated endocytosis and Rab11 Mol Biol Cell 2004 15 4166 4178 10.1091/mbc.E04-03-0245 15229286 PMC515349

[B85] Penheiter SG Singh RD Repellin CE Wilkes MC Edens M Howe PH Pagano RE Leof EB Type II transforming growth factor-beta receptor recycling is dependent upon the clathrin adaptor protein Dab2 Mol Biol Cell 2010 21 4009 4019 10.1091/mbc.E09-12-1019 20881059 PMC2982134

[B86] Ma X Wang Q Jiang Y Xiao Z Fang X Chen YG Lateral diffusion of TGF-beta type I receptor studied by single-molecule imaging Biochem Biophys Res Commun 2007 356 67 71 10.1016/j.bbrc.2007.02.080 17346672

[B87] Luga V McLean S Le Roy C O'Connor-McCourt M Wrana JL Di Guglielmo GM The extracellular domain of the TGFbeta type II receptor regulates membrane raft partitioning Biochem J 2009 421 119 131 10.1042/BJ20081131 19356148

[B88] Zhang XL Topley N Ito T Phillips A Interleukin-6 regulation of transforming growth factor (TGF)-beta receptor compartmentalization and turnover enhances TGF-beta1 signaling J Biol Chem 2005 280 12239 12245 15661740 10.1074/jbc.M413284200

[B89] Atfi A Dumont E Colland F Bonnier D L'Helgoualc'h A Prunier C Ferrand N Clement B Wewer UM Theret N The disintegrin and metalloproteinase ADAM12 contributes to TGF-beta signaling through interaction with the type II receptor J Cell Biol 2007 178 201 208 10.1083/jcb.200612046 17620406 PMC2064440

[B90] Chen CL Huang SS Huang JS Cellular heparan sulfate negatively modulates transforming growth factor-beta1 (TGF-beta1) responsiveness in epithelial cells J Biol Chem 2006 281 11506 11514 10.1074/jbc.M512821200 16492675

[B91] Ito T Williams JD Fraser DJ Phillips AO Hyaluronan regulates transforming growth factor-beta1 receptor compartmentalization J Biol Chem 2004 279 25326 25332 10.1074/jbc.M403135200 15084590

[B92] Razani B Zhang XL Bitzer M von Gersdorff G Bottinger EP Lisanti MP Caveolin-1 regulates transforming growth factor (TGF)-beta/SMAD signaling through an interaction with the TGF-beta type I receptor J Biol Chem 2001 276 6727 6738 10.1074/jbc.M008340200 11102446

[B93] Bizet AA Liu K Tran-Khanh N Saksena A Vorstenbosch J Finnson KW Buschmann MD Philip A The TGF-beta co-receptor, CD109, promotes internalization and degradation of TGF-beta receptors Biochim Biophys Acta 2011 1813 742 753 10.1016/j.bbamcr.2011.01.028 21295082

[B94] Bizet AA Tran-Khanh N Saksena A Liu K Buschmann MD Philip A CD109-mediated degradation of TGF-beta receptors and inhibition of TGF-beta responses involve regulation of SMAD7 and Smurf2 localization and function J Cell Biochem 2011 10.1002/jcb.23349 21898545

[B95] Zuo W Chen YG Specific activation of mitogen-activated protein kinase by transforming growth factor-beta receptors in lipid rafts is required for epithelial cell plasticity Mol Biol Cell 2009 20 1020 1029 19056678 10.1091/mbc.E08-09-0898 PMC2633387

[B96] Bandyopadhyay B Han A Dai J Fan J Li Y Chen M Woodley DT Li W TbetaRI/Alk5-independent TbetaRII signaling to ERK1/2 in human skin cells according to distinct levels of TbetaRII expression J Cell Sci 2011 124 19 24 10.1242/jcs.076505 21172820 PMC3001406

[B97] Lee BI Park SH Kim JW Sausville EA Kim HT Nakanishi O Trepel JB Kim SJ MS-275, a histone deacetylase inhibitor, selectively induces transforming growth factor beta type II receptor expression in human breast cancer cells Cancer Res 2001 61 931 934 11221885

[B98] Ammanamanchi S Brattain MG Restoration of transforming growth factor-beta signaling through receptor RI induction by histone deacetylase activity inhibition in breast cancer cells J Biol Chem 2004 279 32620 32625 10.1074/jbc.M402691200 15155736

[B99] Huang W Zhao S Ammanamanchi S Brattain M Venkatasubbarao K Freeman JW Trichostatin A induces transforming growth factor beta type II receptor promoter activity and acetylation of Sp1 by recruitment of PCAF/p300 to a Sp1.NF-Y complex J Biol Chem 2005 280 10047 10054 10.1074/jbc.M408680200 15647279

[B100] Osada H Tatematsu Y Sugito N Horio Y Takahashi T Histone modification in the TGFbetaRII gene promoter and its significance for responsiveness to HDAC inhibitor in lung cancer cell lines Mol Carcinog 2005 44 233 241 10.1002/mc.20135 16163707

[B101] Wang H Liu J Zong Y Xu Y Deng W Zhu H Liu Y Ma C Huang L Zhang L Qin C miR-106b aberrantly expressed in a double transgenic mouse model for Alzheimer's disease targets TGF-beta type II receptor Brain Res 2010 1357 166 174 20709030 10.1016/j.brainres.2010.08.023

[B102] Wang Q Huang Z Xue H Jin C Ju XL Han JD Chen YG MicroRNA miR-24 inhibits erythropoiesis by targeting activin type I receptor ALK4 Blood 2008 111 588 595 10.1182/blood-2007-05-092718 17906079

